# Walking Ahead: The Headed Social Force Model

**DOI:** 10.1371/journal.pone.0169734

**Published:** 2017-01-11

**Authors:** Francesco Farina, Daniele Fontanelli, Andrea Garulli, Antonio Giannitrapani, Domenico Prattichizzo

**Affiliations:** 1 Dipartimento di Ingegneria dell’Informazione e Scienze Matematiche, Università di Siena, Siena, Italy; 2 Dipartimento di Ingegneria Industriale, Università di Trento, Trento, Italy; Beihang University, CHINA

## Abstract

Human motion models are finding an increasing number of novel applications in many different fields, such as building design, computer graphics and robot motion planning. The Social Force Model is one of the most popular alternatives to describe the motion of pedestrians. By resorting to a physical analogy, individuals are assimilated to point-wise particles subject to social forces which drive their dynamics. Such a model implicitly assumes that humans move isotropically. On the contrary, empirical evidence shows that people do have a preferred direction of motion, walking forward most of the time. Lateral motions are observed only in specific circumstances, such as when navigating in overcrowded environments or avoiding unexpected obstacles. In this paper, the Headed Social Force Model is introduced in order to improve the realism of the trajectories generated by the classical Social Force Model. The key feature of the proposed approach is the inclusion of the pedestrians’ heading into the dynamic model used to describe the motion of each individual. The force and torque representing the model inputs are computed as suitable functions of the force terms resulting from the traditional Social Force Model. Moreover, a new force contribution is introduced in order to model the behavior of people walking together as a single group. The proposed model features high versatility, being able to reproduce both the unicycle-like trajectories typical of people moving in open spaces and the point-wise motion patterns occurring in high density scenarios. Extensive numerical simulations show an increased regularity of the resulting trajectories and confirm a general improvement of the model realism.

## Introduction

There is an indisputable steadily increasing attention on human motion models in different research areas, ranging from building architectural design to service robotic planning and control. A taxonomy of the different approaches proposed in the literature can be found in the survey [1], which describes different models suitable for building evacuation dynamics in both emergency and normal situations. The models proposed for this kind of problems have been historically based on macroscopic quantities, such as densities and fluids [2]. In more standard circumstances, where the interactions are less frequent than in overcrowded evacuation dynamics, a microscopic description of pedestrians is preferable. In the latter case, the proposed approaches can be roughly categorized into four main classes: cellular automata [3], agent-based models [4], graph-based methods [5] and social force models [6]. Cellular automata are especially suitable for modeling human motion in complex environments. This models consist of a discrete system evolving on a discrete set of cells, at discrete time intervals. The value of each cell depends on the modeled behavior of the agent occupying it, on the neighboring cell values and on a set of local updating rules (e.g., see [7–12]). Agent-based approaches model the active and reactive behaviors of the pedestrians according to stochastic models. In this framework, constant velocity models have received large attention since they are easily tractable and allow the direct use of Kalman filters for predictions and belief computations (e.g., see [13, 14]). In graph-based approaches, the environment is subdivided into regions using empirical observations and learning algorithms. The regions are usually mapped as nodes on the graph, while the paths joining them are the arcs. The nodes are usually considered as places in the environment of particular interest, where people stop or make decisions (e.g., see [5, 15]).

The idea of modeling pedestrian motions by using a system of forces describing social interactions dates back to 1979. In [16], magnetic forces acting on a pedestrian and generated by a magnetic pole have been used for computer simulations, with the purpose of designing building architectures. The Social Force Model (SFM) [6, 17] is one of the most popular human motion models based on social forces. In the SFM, each individual is assimilated to a point-wise particle subject to social forces. Hence, the pedestrians’ dynamics are described by means of a system of differential equations. The SFM is especially well suited to reproduce individual motion of pedestrians in high-density scenarios (crowd), as well as the interactions occurring among pedestrians. The potential of the SFM, and in general of models based on social forces, in providing realistic representations of crowd behaviors has been widely acknowledged [18–20]. Due to this, the original formulation of the SFM has been successively refined in the literature. For example, in [21] the authors propose an alternate version considering both relative positions and velocities, which works particularly well for low density cases. Relative velocities between pedestrians are instead considered in [22], while [23] uses pedestrians’ absolute velocities to govern the user head-on interactions. The relative positions and velocities provide also a way to account for the stop situation, which cannot be modeled by the original model [24, 25]. For example, [24] proposes three different SFM models for agents that are standing still. The models describe the possibility of the agent to avoid incoming humans by coding a step forward/backward behavior, the ability to recover its desired position as well as changing it according to the environmental situation. The idea of relative velocities is further extended in [26], where the estimate of the “time to collision” is included in the SFM formulation for repulsive forces. Some versions of the SFM take explicitly into account the prediction of possible collisions, as in [27], where the time to collision is used for lane-like avoidance, or in [28], where an additional force term is added to the original SFM as a function of the body and face poses.

To the best of the authors’ knowledge, the different versions of the SFM have not explicitly modeled the dynamics of the pedestrians’ heading so far. In the literature contributions previously reviewed, a person is supposed to be able to move freely in any direction at any time. On the contrary, empirical evidence shows that, most of the time, pedestrians tend to move forward, i.e. their velocity vector is most often aligned with their heading, due to the biomechanics of humans. This phenomenon has been observed by several studies [29–31], which come to the conclusion that a *nonholonomic* model may be more appropriate to describe human motion in many cases. For instance, unicycle-like models, widely used in the mobile robotics field, are able to accurately reproduce goal-oriented locomotion of an individual moving in free space [29]. Moreover, the adoption of such models in [30] allow the authors to give a nice interpretation of the mechanism underlying the formation of human trajectories (namely, the minimization of the time derivative of the path curvature).

In this paper, we introduce the Headed Social Force Model (HSFM) in order to enhance the traditional SFM by explicitly accounting for the pedestrians’ heading. To this end, we describe the motion of each individual by means of a dynamic model similar to that adopted in [32] for generating biologically-inspired robot trajectories. The contribution of the paper is twofold. First, we propose a new method for generating the forces and torques driving the dynamics of each pedestrian, with the purpose of maximizing the realism of the resulting trajectories. In doing so, several conflicting objectives have to be taken into account. In low density scenarios, the pedestrians’ motion should be as smooth as possible, consistently with what is empirically observed [33]. In these circumstances, lateral motions should be avoided because individuals walk ahead most of the time. On the contrary, in crowded or cluttered environments, the interaction among pedestrians, as well as between pedestrians and the environment, is stronger and determines most of the pedestrians’ trajectories. The proposed solution consists in computing the model inputs as suitable functions of the force terms adopted in the traditional SFM. The second contribution of the paper is the introduction of an additional force in order to reproduce the behavior of people intentionally walking together as a single group (e.g., friends or colleagues). This is achieved by defining a desired region (depending on both the position and the heading of the pedestrians) within which the group is expected to lie as a result of the social ties among group members. The new force term is designed to drive the individuals back into the region whenever they leave it. This allows the model to rule out trajectories which do not facilitate social interaction, such as pedestrians arranged in a single line or spread over large areas. It is shown that the introduction of the preference towards nonholonomic motions in the proposed model does not compromise its ability to reproduce individuals moving in groups. Overall, considering the pedestrians’ heading enhances the fidelity of the model in two ways. Whenever nonholonomic motion patterns naturally arise, the generated trajectories resemble more closely those empirically observed. Typical examples include people walking in open spaces or reaching close targets. More generally, accounting explicitly for the pedestrians’ heading helps to increase the regularity of the trajectories, resulting in fewer abrupt changes of direction and a reduced number of collisions. The performance of the HSFM is evaluated via numerical simulation under very different operating conditions, and a sensitivity analysis of the model behavior with respect to variations in the model parameters is presented. As a byproduct, guidelines for the selection of the parameter values are obtained.

The paper is structured as follows. The proposed model is presented in the next section. Then, numerical results illustrating the model behavior in three different scenarios are presented and discussed. Finally, some conclusions are drawn.

## The Headed Social Force Model

Humans walk ahead most of the time, and their motion can be well approximated by nonholonomic models [29]. There are some circumstances, though, in which sideward motions violating nonholonomic constraints, are commonly observed (e.g., avoiding unexpected obstacles, negotiating a narrow passage or navigating in highly crowded places). In these cases, a *holonomic* model is preferable (with a slight abuse of terminology, here we denote by “holonomic model” any model not subject to nonholonomic constraints, thus including unconstrained models). In order to account for such a variability, in the HSFM each individual is modeled by means of a dynamic system like that presented in [32], which is able to reproduce both holonomic and nonholonomic motion patterns by suitably designing the system inputs (i.e., the forces and torques driving the dynamics of the pedestrians’ position and heading). In the HSFM, such inputs are designed as suitable functions of the social forces acting on each individual, computed according to the traditional SFM. Let

$$\begin{array}{c} f_{i}=f_{i}^{0}+f_{i}^{e} \end{array}$$ (1)

denote the total force acting on individual *i* according to the SFM. The term $f_{i}^{0}$ is the force attracting the pedestrian towards her target, such as a waypoint, whereas $f_{i}^{e}$ accounts for repulsive and interaction forces among individuals, and between individuals and the environment. In a sense, $f_{i}^{0}$ models long-term objectives, such as travelling a prescribed path, whereas the force terms in $f_{i}^{e}$ account for short-term corrective actions, such as maneuvers needed to avoid nearby obstacles or pedestrians. Then, in the HSFM, the motion of pedestrians is generated as follows.

**Fig 1:**
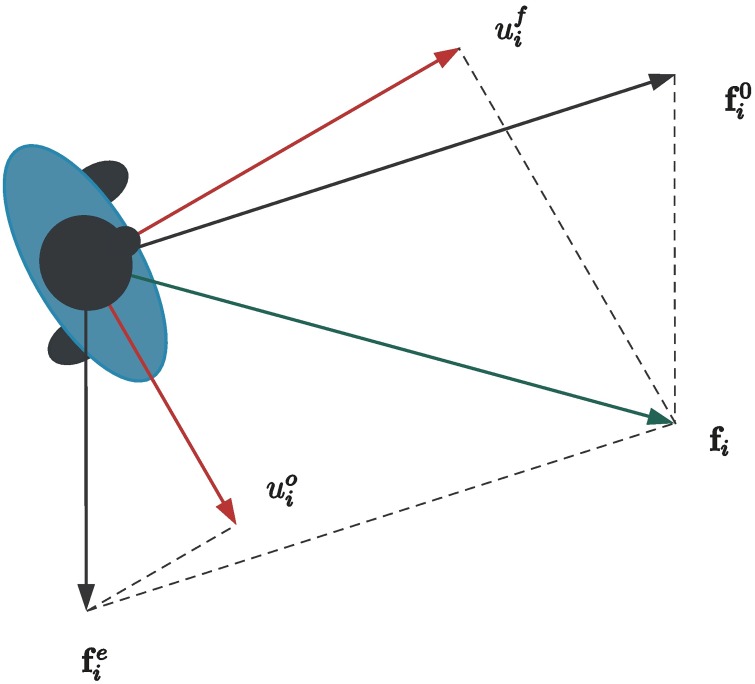
Force decomposition in the Headed Social Force Model. The force $u_{i}^{f}$, acting along the forward direction, is the projection (along the same direction) of the total force **f**_*i*_ resulting from the traditional SFM. The force $u_{i}^{o}$, acting along the orthogonal direction, is the projection (along the same direction) of the $f_{i}^{e}$ force alone.

- The forces $u_{i}^{f}$ and $u_{i}^{o}$ driving the *translational dynamics* are computed by projecting **f**_*i*_ and $f_{i}^{e}$ along the forward direction of motion (identified by the pedestrian’s heading) and the orthogonal direction of motion, respectively (see Fig 1).
- The torque driving the *rotational dynamics* is proportional to the projection of the term $f_{i}^{0}$ along the orthogonal direction of motion.
- An additional force term is added in order to ensure *group cohesion* when simulating people moving together. This is achieved by: i) defining a rectangular region, centered at the group centroid, within which the group members are expected to lie, and ii) exerting a force pushing the pedestrians back into that region whenever they get out of it.

In both the translational and the rotational dynamics, damping terms are included in order to weaken oscillations and obtain smoother trajectories. Only the force $f_{i}^{e}$ is assumed to affect lateral moves, because they are mainly caused by the interactions with other pedestrians or the environment. On the other hand, body rotations are generated by the lateral component of the force $f_{i}^{0}$, which is in charge of driving the pedestrian towards the goal. The rationale behind this choice is that a person will tend to turn faster towards the target, the more she is attracted by the target itself. The idea of the group cohesion force is inspired by the approach proposed in [34] for modeling small groups of pedestrians (from two to four individuals) walking together and subject to social interaction constraints. In this paper, such an approach is adapted to the proposed dynamic model which accounts for pedestrians’ heading. In particular, the force term is designed in order to reproduce the formation of larger groups, including many individuals moving together (e.g., like a group of tourists following a guide).

The proposed HSFM enriches the traditional SFM with a more complex human locomotion model which is well suited to represent human trajectories complying with nonholonomic constraints, as typically occurs in large spaces occupied by a limited number of pedestrians. At the same time, the HSFM preserves the power of the SFM in realistically reproducing the flow of a large number of people moving in densely populated environments. A unique feature of the proposed model lies in its ability to adapt to the external conditions, by smoothly switching between holonomic and nonholonomic motion patterns depending on a number of factors, including the pedestrian density, the pedestrians’ goal and the clutter of the environment. Notably, this behavior is achieved without the need of changing online any of the model parameters, but as a natural reaction and adaptation to the external conditions. In the next section, we will show some examples of trajectories generated according to the HSFM in order to highlight: i) the different behavior with respect to the SFM in specific circumstances; ii) the preservation of the nice features possessed by the SFM; iii) the ability of reproducing the motion of group of people walking together. In the remaining of this section, the details of the proposed model are presented.

### Dynamic Model

Consider a system of *n* pedestrians moving in a 2D environment. Following the modeling approach of the Social Force Model [6, 17], the *i*-th individual, *i* = 1, …, *n*, is assimilated to a particle with mass *m*_*i*_, whose position and velocity, expressed in a global reference frame, are denoted by **r**_*i*_ = [*x*_*i*_, *y*_*i*_]^⊤^ and $v_{i}={\left[{\dot{x}}_{i},\;{\dot{y}}_{i}\right]}^{⊤}$, respectively. The equations of motion are

$$\begin{array}{l} {\dot{r}}_{i}=v_{i},\\ {\dot{v}}_{i}=\frac{1}{m_{i}}u_{i}, \end{array}$$

where **u**_*i*_ represents the social force driving the *i*-th particle. In order to include the pedestrians’ heading into the model, it is convenient to attach a body frame to each individual, i.e. a reference frame centered at the pedestrian’s position and whose *x*-axis is aligned with the pedestrian’s forward direction of motion. Let **q**_*i*_ = [*θ*_*i*_, *ω*_*i*_]^⊤^ be the vector containing the heading *θ*_*i*_ (angle between the *x*-axis of the body frame and that of the global reference frame) and the angular velocity $\omega_{i}={\dot{\theta }}_{i}$ of the *i*-th pedestrian. Denote by $v_{i}^{B}={\left[v_{i}^{f},\;v_{i}^{o}\right]}^{⊤}$ the velocity vector expressed in the body frame. The components $v_{i}^{f}$ and $v_{i}^{o}$ of vector $v_{i}^{B}$ correspond to the projection of the velocity vector **v**_*i*_ along the forward direction and the orthogonal direction, respectively. Clearly, $v_{i}=\text{R}(\theta_{i}){\text{v}}_{i}^{B}$ where the rotation matrix **R**(*θ*_*i*_) is defined as

$$\begin{array}{c} R\left(\theta_{i}\right)=\left[\begin{array}{cc} \cos(\theta_{i}) & -\sin(\theta_{i})\\ \sin(\theta_{i}) & \cos(\theta_{i}) \end{array}\right]≐\left[r_{i}^{f}\;r_{i}^{o}\right]. \end{array}$$

Then, similarly to [32], the human locomotion model can be written as

$${\dot{r}}_{i}=R\left(\theta_{i}\right)v_{i}^{B},$$ (2)

$${\dot{v}}_{i}^{B}=\frac{1}{m_{i}}u_{i}^{B},$$ (3)

$${\dot{q}}_{i}=Aq_{i}+b_{i}u_{i}^{\theta },$$ (4)

where

$$\begin{array}{c} A=\left[\begin{array}{cc} 0 & 1\\ 0 & 0 \end{array}\right],\;b_{i}=\left[\begin{array}{c} 0\\ \frac{1}{I_{i}} \end{array}\right], \end{array}$$ (5)

and *I*_*i*_ denotes the moment of inertia of pedestrian *i*. In models (2)–(4), the inputs are $u_{i}^{B}={\left[u_{i}^{f},\;u_{i}^{o}\right]}^{⊤}$, whose entries are the forces acting along the forward direction and the orthogonal direction, respectively, as well as the torque $u_{i}^{\theta }$ about the vertical axis. Notice that such a model is indeed unconstrained. However, if $v_{i}^{o}\left(0\right)=0$ and $u_{i}^{o}\left(t\right)=0$, for all *t*, the dynamic unicycle model is recovered. In general, whenever $v_{i}^{o}=0$, the model features a nonholonomic behavior, the velocity vector being aligned with the pedestrian’s heading.

The key idea of the HSFM is to compute the model inputs $u_{i}^{f}$, $u_{i}^{o}$ and $u_{i}^{\theta }$ on the basis of the forces resulting from the traditional SFM. To this purpose, the total force **f**_*i*_ that acts on the *i*-th pedestrian according to [17] is decomposed as in Eq (1). The first term

$$\begin{array}{c} f_{i}^{0}=m_{i}\frac{v_{i}^{d}-v_{i}}{\tau_{i}} \end{array}$$ (6)

accounts for the pedestrian’s desire to move with a given velocity vector $v_{i}^{d}$. In Eq (6), the characteristic time *τ*_*i*_ > 0 is a parameter determining the rate of change of the velocity vector. The second force term

$$\begin{array}{c} f_{i}^{e}=f_{i}^{p}+f_{i}^{w} \end{array}$$ (7)

accounts for the pedestrians’ interaction. The terms $f_{i}^{p}$ and $f_{i}^{w}$ represent the repulsive forces exerted on individual *i* by the other pedestrians and by possible obstacles present in the environment (e.g., walls), respectively. The expressions of $f_{i}^{p}$ and $f_{i}^{w}$ are reported for completeness at the end of this section. The inputs of the HSFM are computed from $f_{i}^{0}$ and $f_{i}^{e}$ as follows.

### Force Input

The input vector $u_{i}^{B}$ includes the forces acting along the pedestrian’s forward direction and the orthogonal direction. Given the total social force **f**_*i*_, a natural choice for computing $u_{i}^{f}$ is to project **f**_*i*_ along the forward direction. In order to avoid sideward motions if not strictly needed, the component $u_{i}^{o}$ is computed by projecting the interaction force $f_{i}^{e}$ (possibly scaled), along the orthogonal direction. Finally, in order to drive to zero the sideward velocity $v_{i}^{o}$ when the sideward force is zero, a damping term proportional to $v_{i}^{o}$ is added to $u_{i}^{o}$. Hence, the model inputs $u_{i}^{f}$ and $u_{i}^{o}$ are computed as

$$u_{i}^{f}={\left(f_{i}^{0}+f_{i}^{e}\right)}^{⊤}r_{i}^{f},$$ (8)

$$u_{i}^{o}=k^{o}{\left(f_{i}^{e}\right)}^{⊤}r_{i}^{o}-k^{d}v_{i}^{o},$$ (9)

where *k*^*o*^ > 0 and *k*^*d*^ > 0.

### Torque Input

The input $u_{i}^{\theta }$ represents the torque about the vertical axis which drives the dynamics of the pedestrian’s heading. This term is designed on the basis of the force $f_{i}^{0}$ defined in Eq (6). Denote by $f_{i}^{0}$ and $\theta_{i}^{0}$ the magnitude and the phase in the global reference frame of $f_{i}^{0}$. Notice that both quantities are in general time-varying. The input $u_{i}^{\theta }$ is computed as

$$\begin{array}{c} u_{i}^{\theta }=-k^{\theta }\left(\theta_{i}-\theta_{i}^{0}\right)-k^{\omega }\omega_{i}. \end{array}$$ (10)

The parameters *k*^*θ*^ and *k*^*ω*^ are designed in order to achieve suitable dynamics of the heading. It can be easily verified that, with $u_{i}^{\theta }$ defined as in Eq (10), the orientation error ${\tilde{\theta }}_{i}≐\theta_{i}-\theta_{i}^{0}$ evolves according to the dynamic model

$$\begin{array}{c} {\ddot{\tilde{\theta }}}_{i}+\frac{k^{\omega }}{I_{i}}{\dot{\tilde{\theta }}}_{i}+\frac{k^{\theta }}{I_{i}}{\tilde{\theta }}_{i}=-\frac{k^{\omega }}{I_{i}}{\dot{\theta }}_{i}^{0}-{\ddot{\theta }}_{i}^{0}. \end{array}$$ (11)

A possible design procedure is to select the values of *k*^*θ*^ and *k*^*ω*^ on the basis of the desired poles *λ*_1_ and *λ*_2_ of the dynamic system Eq (11). In this work, real poles are considered, so that *λ*_2_ = *αλ*_1_ < 0, for some *α* > 1. In turn, the dominant pole *λ*_1_ is selected as a function of $f_{i}^{0}$

$$\begin{array}{c} \lambda_{1}=-\sqrt{\frac{k^{\lambda }f_{i}^{0}}{\alpha }}, \end{array}$$

where *k*^*λ*^ > 0 is used to tune the dominant time constant of system Eq (11). The corresponding expressions of *k*^*θ*^ and *k*^*ω*^ are then

$$\begin{array}{c} k^{\theta }=I_{i}k^{\lambda }f_{i}^{0},\;k^{\omega }=I_{i}\left(1+\alpha \right)\sqrt{\frac{k^{\lambda }f_{i}^{0}}{\alpha }}. \end{array}$$ (12)

Choosing the poles *λ*_1_ and *λ*_2_ as functions of $f_{i}^{0}$ allows one to modulate the responsiveness of the system with the intensity of the driving force. The underlying idea is that the more authoritative the $f_{i}^{0}$, the faster the change in the pedestrian’s heading. In this way, the heading convergence rate is proportional to $f_{i}^{0}$.

### Group Cohesion

In order to model a group of people moving together, the force input Eqs (8) and (9) can be modified by adding an additional term, which forces the pedestrians to lie within a given box. Let $c=\frac{1}{n}\sum_{j=1}^{n}r_{i}$ be the centroid of the group, and define **p**_*i*_ = **c** − **r**_*i*_. The model inputs $u_{i}^{f}$ and $u_{i}^{o}$ are computed as

$$u_{i}^{f}={\left(f_{i}^{0}+f_{i}^{e}\right)}^{⊤}r_{i}^{f}+k_{1}^{g}h\left(p_{i},r_{i}^{f},d^{f}\right),$$ (13)

$$u_{i}^{o}=k^{o}{\left(f_{i}^{e}\right)}^{⊤}r_{i}^{o}-k^{d}v_{i}^{o}+k_{2}^{g}h\left(p_{i},r_{i}^{o},d^{o}\right),$$ (14)

where $k_{1}^{g}>0$, $k_{2}^{g}>0$ and

$$\begin{array}{c} h\left(x,y,z\right)=\left\{\begin{array}{cc} 1 & \text{if}\;|x^{⊤}y|>z\\ 0 & \text{otherwise}. \end{array}\right. \end{array}$$ (15)

The parameters *d*^*f*^ > 0 and *d*^*o*^ > 0 denote the semilength of the box sides.

### SFM Force expressions

The expressions of $f_{i}^{p}$ and $f_{i}^{w}$ in Eq (7) are taken from [17]. Let the radius of the *i*-th pedestrian be denoted by *r*_*i*_. Moreover, let us define

$$\begin{array}{l} r_{ij}=r_{i}+r_{j},\\ d_{ij}=∥r_{i}-r_{j}∥, \end{array}$$ (16)

$$n_{ij}=\frac{r_{i}-r_{j}}{∥r_{i}-r_{j}∥}≐{\left[n_{ij}\left(1\right),n_{ij}\left(2\right)\right]}^{\prime },$$ (17)

$$t_{ij}={\left[-n_{ij}\left(2\right),n_{ij}\left(1\right)\right]}^{\prime },$$ (18)

$$\Delta v_{ij}^{\left(t\right)}={\left(v_{j}-v_{i}\right)}^{\prime }t_{ij}.$$ (19)

- The term $f_{i}^{p}$, modeling the repulsive effects of other pedestrians on individual *i*, is given by $f_{i}^{p}=\sum_{j,\;j\neq i}f_{ij}^{p}$. The force exerted by pedestrian *j* on pedestrian *i* is
  $$\begin{array}{l} f_{ij}^{p}=\left[A_{i}e^{\left(r_{ij}-d_{ij}\right)/B_{i}}+k_{1}g\left(r_{ij}-d_{ij}\right)\right]n_{ij}\\ \begin{array}{cc} & \;+k_{2}g\left(r_{ij}-d_{ij}\right)\Delta v_{ij}^{\left(t\right)}t_{ij}, \end{array} \end{array}$$ (20)
  where *g*(*x*) = max{0, *x*} and *A*_*i*_, *B*_*i*_, *k*_1_ and *k*_2_ are constant parameters. Notice that **f**_*ij*_ is composed by three terms. The first one, $A_{i}e^{\left(r_{ij}-d_{ij}\right)/B_{i}}n_{ij}$, represents the repulsive term, while *k*_1_ *g*(*r*_*ij*_ − *d*_*ij*_)**n**_*ij*_ and $k_{2}g\left(r_{ij}-d_{ij}\right)\Delta v_{ij}^{\left(t\right)}t_{ij}$ represent the compression and friction forces, respectively, and come into play only if *d*_*ij*_ < *r*_*ij*_.
- The term $f_{i}^{w}$, modeling the repulsive effects of obstacles or boundaries such as walls on individual *i*, is given by $f_{i}^{w}=\sum_{w}f_{iw}^{w}$. The force exerted by wall *w* on pedestrian *i* is
  $$\begin{array}{l} f_{iw}^{w}=\left[A_{w}e^{\left(r_{i}-d_{iw}\right)/B_{w}}+k_{1}g\left(r_{i}-d_{iw}\right)\right]n_{iw}\\ \begin{array}{cc} & \;-k_{2}g\left(r_{i}-d_{iw}\right)\Delta v_{iw}^{\left(t\right)}t_{iw}. \end{array} \end{array}$$ (21)
  The expression of $f_{iw}^{w}$ is is pretty similar to that of the repulsive force between pedestrians $f_{ij}^{p}$. Quantities *d*_*iw*_, **n**_*iw*_, **t**_*iw*_ and $\Delta v_{iw}^{\left(t\right)}$ are defined according to Eqs (16)–(19), by replacing **r**_*j*_ with the coordinates of the closest point of wall *w* to pedestrian *i* and setting **v**_*j*_ = 0.

## Results and Discussion

In this section, the results of a number of numerical simulations are reported, in order to highlight the characteristic features of the proposed model. Three different scenarios are considered. In Scenario I, we simulate two simple case studies, involving a single pedestrian, aimed at showing the high fidelity of the HSFM in reproducing the trajectories of pedestrians moving in free space according to a nonholonomic behavior. In Scenario II, we consider three different experiments, involving a number of pedestrians ranging from 20 to 200. The purpose is to illustrate the ability of the HSFM to automatically adapt the generated trajectories to the external context, smoothly relaxing the nonholonomic constraints as the pedestrian density increases or unexpected obstacles come into play. In Scenario III, we consider a more articulated case study, by simulating a group of 10 people visiting a museum together. The focus of this study is to show how the group force introduced in the HSFM originates trajectories preserving the cohesion of the group. The section ends with a discussion on the role played by the parameters of the HSFM. An extensive simulation campaign is performed in order to analyze the effect of parameter variations on the generated trajectories, thus providing useful guidelines for the tuning of the model.

In all the simulations presented hereafter, the reference velocity vector $v_{i}^{d}$, which is used by the SFM to compute the force $f_{i}^{0}$ (see Eq (6)), is generated as $v_{i}^{d}=v^{d}e_{i}^{d}$. The desired speed *v*^*d*^ is assumed constant over each simulation run. The unit vector $e_{i}^{d}$, which identifies the desired direction of motion, is computed from a sequence of way-points encoding the desired pedestrian path, similarly to [6]. The following values of the HSFM parameters have been used. The radius *r*_*i*_ and the mass *m*_*i*_ of each pedestrian have been randomly generated in the intervals [0.25 m, 0.35 m] and [60 kg, 90 kg], respectively, assuming uniform distributions. The inertia moment *I*_*i*_ in Eq (5) is computed as $I_{i}=\frac{1}{2}m_{i}r_{i}^{2}$, i.e., the pedestrian is assimilated to a cylinder rotating about its main axis. The following parameters entering in the computation of the model inputs Eqs (8)–(12) and (13)–(15) have been used in all the simulations (unless differently stated): *k*^*o*^ = 1, *k*_*d*_ = 500 kg ⋅ s^−1^, *α* = 3, *k*^*λ*^ = 0.3 N^−1^s^−2^, *d*^*f*^ = 2 m, *d*^*o*^ = 1 m and $k_{1}^{g}=k_{2}^{g}=200$ N. The values of the parameters used in the SFM, taken from [17], are: *τ*_*i*_ = 0.5s, *A*_*i*_ = *A*_*w*_ = 2 ⋅ 10^3^N, *B*_*i*_ = *B*_*w*_ = 0.08m, $k_{1}=1.2\cdot 10^{5}\;\text{kg}\;{\text{s}}^{-2}$, $k_{2}=2.4\cdot 10^{5}\;\text{kg}\;{\text{m}}^{-1}{\text{s}}^{-1}$. Videos of the simulations are available at http://control.dii.unisi.it/MobileRoboticsPage.

### Scenario I: The Nonholonomic Behavior

Empirical evidence shows that when a single pedestrian is moving in an open space, she tends to move as a unicycle [29]. To evaluate how well the HSFM can reproduce such a nonholonomic behavior, we consider two use cases.

**Fig 2:**
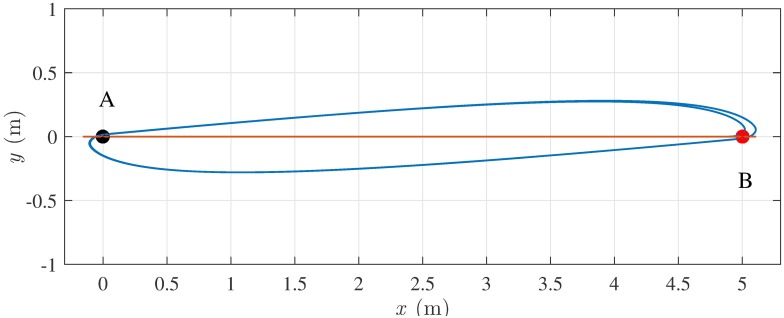
Scenario I, alternate motion between two points. A single pedestrian has to move back and forth between *A* and *B*, starting from *A*, with a desired speed *v*^*d*^ = 1.5 ms^−1^: SFM (red) and HSFM (blue).

In the first example, a single pedestrian walks between two points *A* and *B*, alternately. In this case, the trajectory resulting from the SFM is quite unnatural, as the path boils down to a segment (red line in Fig 2). This phenomenon is due to the SFM neglecting the information about the pedestrian’s heading, so that forward or backward motions are equivalent. On the contrary, the trajectory generated by the HSFM is more realistic thanks to the existence of a preferred direction of motion (blue line in Fig 2). Although the HSFM allows a pedestrian to have her velocity vector not aligned with her heading, the model input tends to drive the orthogonal component of the velocity to zero if no lateral forces are present, thus generating an “almost nonholonomic” behavior. It can be observed that in the resulting path, the pedestrian approaches the turning point preparing to invert her orientation with a sort of U-turn, as it happens in practice.

**Fig 3:**
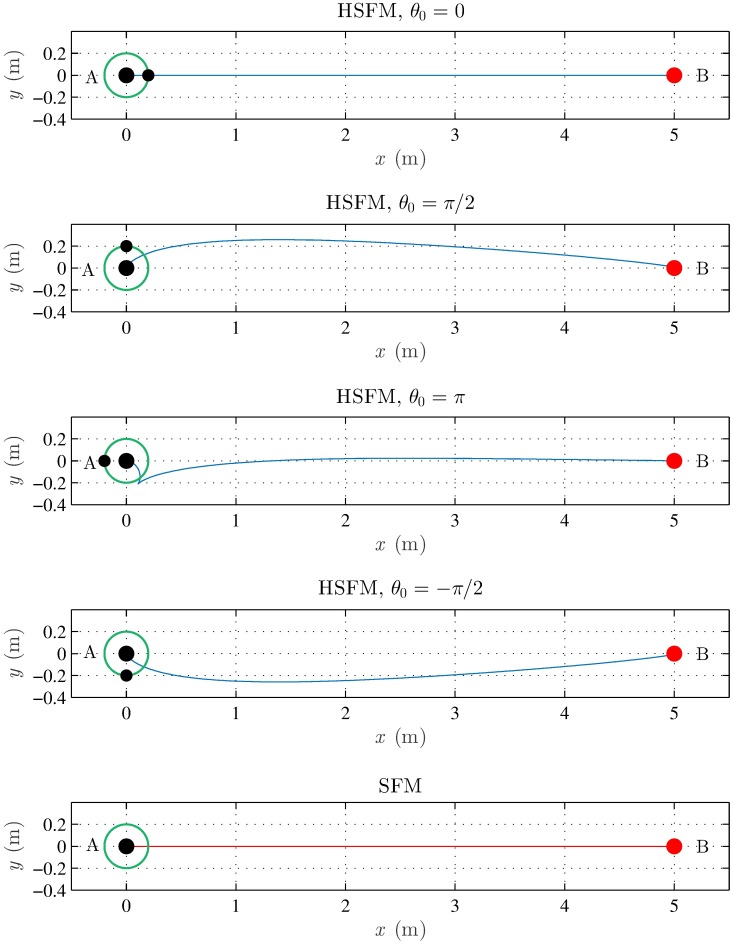
Scenario I, starting with different orientations. A single pedestrian has to move from *A* to *B*, starting with different headings (denoted by the small black dot), at a desired speed *v*^*d*^ = 1.5 ms^−1^: SFM (red) and HSFM (blue).

In the same setting, consider the case in which a pedestrian has to move from *A* to *B*, starting with four different values of the initial heading *θ*(0) (see Fig 3). When *θ*(0) = *π*, the goal point *B* lies behind the pedestrian’s back. In this case, the HSFM makes the pedestrian first take a step back to turn towards the goal, and then move forward to reach the target. Clearly, the SFM trajectory lies on a segment once again, since the heading is neglected.

The previous examples confirm that, in the considered scenario, the HSFM gives rise to a more realistic behavior, endowing the pedestrians with the ability of moving in a nonholonomic way when they are expected to do so, as experimentally verified in [29, 30].

### Scenario II: The Adaptive Behavior

**Fig 4:**
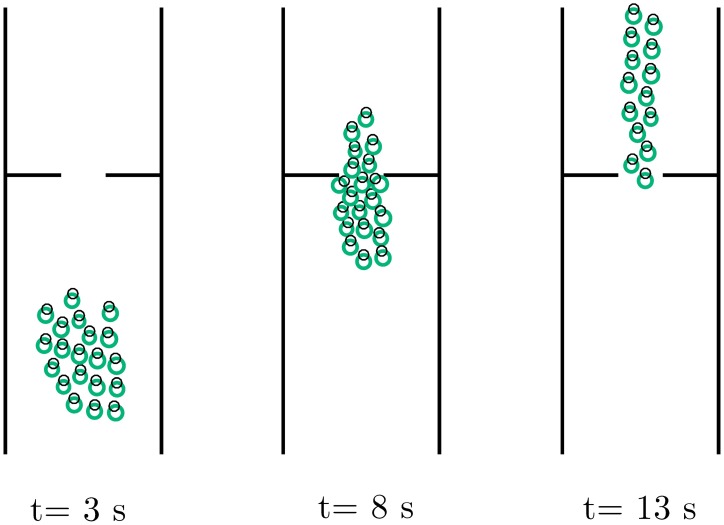
Scenario II, Pedestrians in a corridor. A group of 20 pedestrians walking in the same direction in a 7.5m-wide corridor at a desired speed *v*^*d*^ = 1.5 ms^−1^. Three snapshots of a simulation run of the HSFM, taken at different time instants *t*.

**Fig 5:**
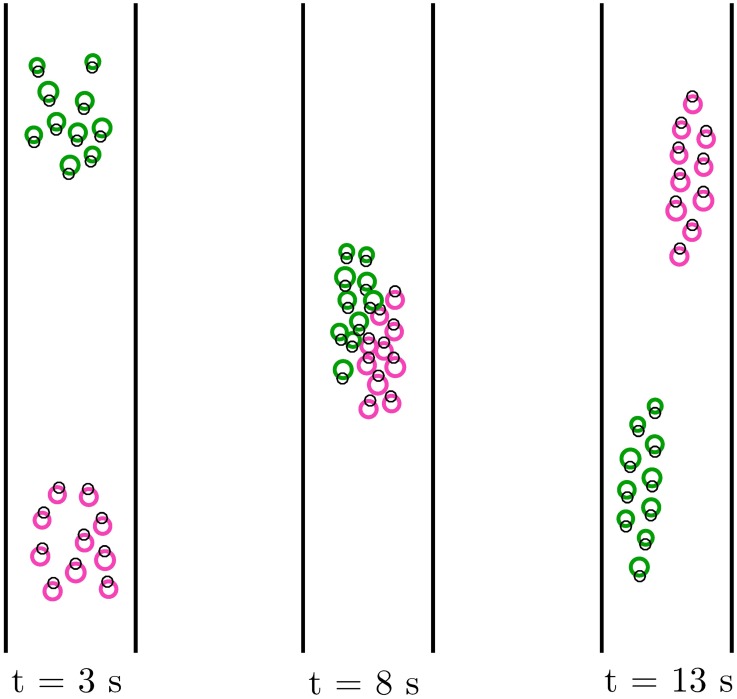
Scenario II, Two groups walking in opposite directions. Two groups of 10 pedestrians each walking in opposite directions in a 5m-wide corridor at a desired speed *v*^*d*^ = 1.5 ms^−1^. Three snapshots of a simulation run of the HSFM, taken at different time instants *t*.

In this scenario, we consider three examples. In the first one, 20 pedestrians walking in a 7.5m-wide corridor have to pass through a 2m-wide door (see Fig 4). In the second example, two groups of pedestrians are walking in opposite directions in a 5m-wide corridor (see Fig 5). In the third example, we simulate passengers boarding on a metro train, similarly to what has been done in [35] to analyze pedestrian counter flow through a bottleneck.

For comparison purposes, the following indicators are considered:

- the average *exit frequency* of pedestrians $\bar{F}$, i.e. the average number of pedestrians that pass through the door per time unit (first and third examples);
- the average *square of the magnitude of the jerk* of the trajectories
  $$\begin{array}{c} \bar{J}=\frac{1}{n}\sum_{i=1}^{n}\frac{1}{T}\int_{0}^{T}\left|\right|j_{i}\left(t\right){\left|\right|}^{2}dt, \end{array}$$ (22)
  where *n* denotes the total number of pedestrians and **j**_*i*_ is the jerk vector of the *i*-th trajectory, (i.e., the third-order derivative of the position).

The first indicator has been selected as a measure of the macroscopic behavior of the models. The second indicator is used to evaluate both the regularity and the realism of the resulting trajectories. As a matter of fact, it is commonly acknowledged that the motions performed by humans tend to be smooth and to minimize the jerk, as first experimentally verified for hand movements in [36], and extended to the trajectories of walking pedestrians later on in [33]. The obtained results can be summarized as follows.

#### Pedestrians in a corridor

In order to compare the trajectories generated by the SFM and the HSFM, a Monte Carlo analysis has been performed. Starting from random initial positions and headings of the pedestrians (with zero initial velocity), 100 runs of the SFM and the HSFM have been simulated for 20 s. Concerning the exit frequency, both models give similar results, with average values ${\bar{F}}_{HSFM}=2.70$ s^−1^ and ${\bar{F}}_{SFM}=2.75$ s^−1^. Overall the two models seem to reproduce the same macroscopic behavior. However, significant differences can be appreciated by looking at the regularity of the resulting trajectories. The average square of the magnitude of the jerk is very different in the two cases, with average values during the door crossing (time range [6, 10] seconds) of ${\bar{J}}_{HSFM}=4.1\cdot 10^{-4}$ m^2^s^−6^ and ${\bar{J}}_{SFM}=5.3\cdot 10^{-3}$ m^2^s^−6^. These figures capture the different qualitative behaviors that can be observed by looking at the resulting trajectories. When compared to the HSFM, in the proximity of the door, the SFM tends to generate vibrations, sudden changes of direction and even “bounces” among pedestrians or between pedestrians and walls.

#### Two groups walking in opposite directions

**Fig 6:**
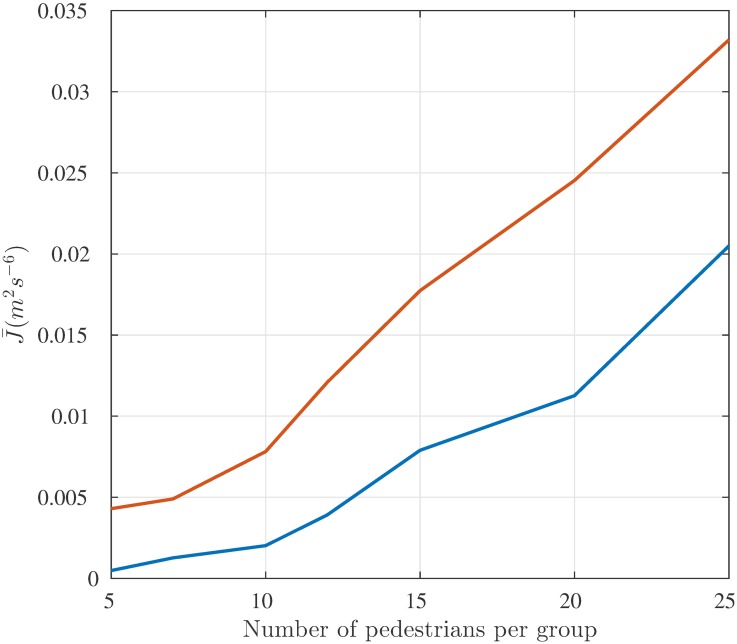
Scenario II, Values of $\bar{J}$ for different pedestrian densities. Average jerk ${\bar{J}}_{HSFM}$ (blue) and ${\bar{J}}_{SFM}$ (red) for two groups of *N* pedestrians each, with *N* ranging from 5 to 25, walking in opposite directions in a 5m-wide corridor, at a desired speed *v*^*d*^ = 1.5 ms^−1^.

Also in this case, results are averaged over 100 simulation runs. In this example, the huge difference in the values of index $\bar{J}$ (${\bar{J}}_{HSFM}=4.3\cdot 10^{-3}$ m^2^s^−6^ for the HSFM vs. ${\bar{J}}_{SFM}=2.3\cdot 10^{-2}$ m^2^s^−6^ for the SFM) is mostly due to the very different trajectories over the time range [6, 10] seconds, when the two groups interact to negotiate the traversing of the corridor. In this situation, the pedestrian motion generated by the HSFM is much more regular than that reproduced by the SFM, in which several collisions among pedestrians belonging to different groups are experienced. The effect of pedestrian density on the indicator $\bar{J}$ has also been evaluated. Both ${\bar{J}}_{HSFM}$ and ${\bar{J}}_{SFM}$ have been computed for groups of different cardinalities, ranging from 5 to 25 (see Fig 6). As expected, as the density increases, the trajectories tend to be more irregular for both models. However, the HSFM confirms its superiority irrespective of the number of pedestrians.

#### Pedestrian counter flow through a bottleneck

**Fig 7:**
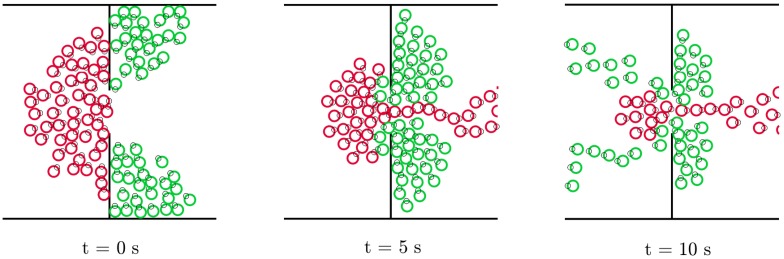
Scenario III, Pedestrian counter flow through a bottleneck. Simulation of a metro train boarding process [35]. Pedestrians in red want to get off the train (towards the right), while pedestrians in green are trying to get on it (towards the left). Three snapshots taken at different time instants.

This example is taken from [35]. Two groups made up of 25 pedestrians each, have to get on board a metro train through a 2m-wide door. Simultaneously, 50 pedestrians are trying to get off the train through the same door (see Fig 7). When simulating such a high density scenario, both the SFM and the HSFM produce a deadlock effect, with the two groups pushing each other in front of the door. In [35], a revised version of the SFM has been presented in order to improve the pedestrians’ efficiency of getting through the bottleneck. The repulsive forces in the SFM have been modified by adding a term which produces a repulsive force in the tangential direction, in order to let a pedestrian slide laterally as she faces another person. These revised forces can be embedded directly in the HSFM, by using them in place of Eqs (20) and (21). The trajectories resulting from the modified versions of both the SFM and the HSFM have been compared. Similar results are obtained in terms of exit frequency for the two models, with average values ${\bar{F}}_{HSFM}=3.16$ s^−1^ and ${\bar{F}}_{SFM}=3.15$ s^−1^. However, the magnitude of the jerk is again different for the two models, with ${\bar{J}}_{HSFM}=0.205\cdot 10^{-1}$ m^2^s^−6^ and ${\bar{J}}_{SFM}=0.42\cdot 10^{-1}$ m^2^s^−6^. This example highlights the versatility of the proposed approach. Other SFM alternative versions, devised for tackling specific scenarios, can be easily incorporated in the HSFM by replacing the original force terms with the modified ones.

Overall, previous results show that at a microscopic level, the HSFM generates smoother trajectories than the traditional SFM. At the same time, the macroscopic behavior of the whole system, which is typically well approximated by the SFM, is fully preserved.

### Scenario III: A Visit at the Museum

**Fig 8:**
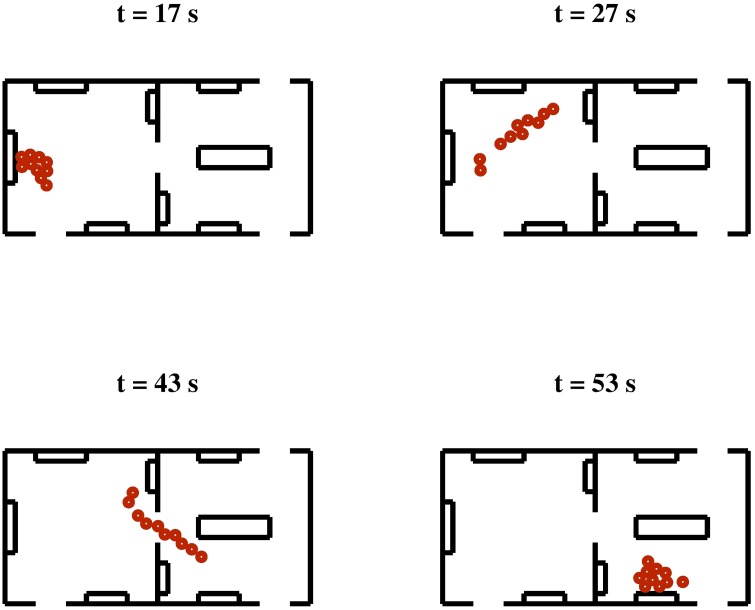
Scenario III, A visit at the Museum. Snapshots of a simulation run of the HSFM without the inclusion of group cohesion forces.

**Fig 9:**
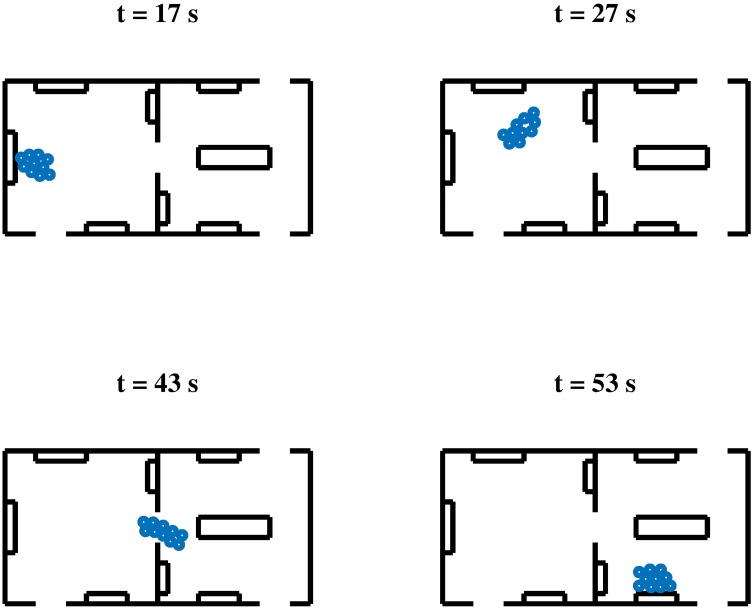
Scenario III, A visit at the Museum. Snapshots of a simulation run of the HSFM with the inclusion of group cohesion forces.

In this scenario we test the ability of the HSFM to reproduce pedestrians moving together. As a case study, we consider the visit of a museum carried out by a group of 10 people. The considered environment is composed of two communicating rooms, each of which contains four artworks on display. Three doors connect the rooms with the rest of the museum (see Figs 8 and 9). The objective of the group is to visit a selection of the pieces of the exhibition in a given order, while avoiding collisions with obstacles and/or other individuals. Once the visitors reach the selected artwork, they stop in front of it for a predefined amount of time, before moving to the next point of interest.

**Fig 10:**
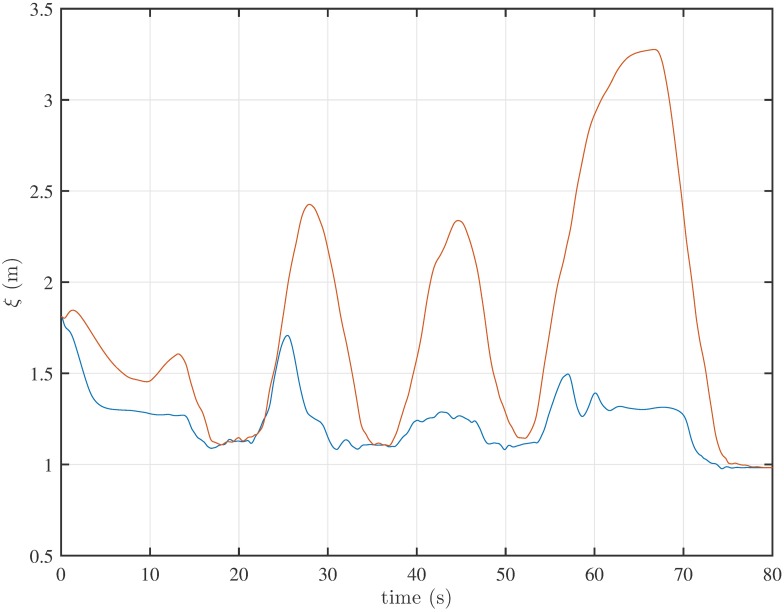
Mean distance from the group centroid over time. Evolution of *ξ* with cohesive forces (blue) and without cohesive forces (red).

We compare the results obtained using the HSFM with and without the group forces. In Figs 8 and 9 four different snapshots of the trajectories from the two cases are shown. The main difference lies in the way the group moves from one exhibition to the other. In the absence of group cohesion forces, the group tends to elongate and the visitors form a line (see Fig 8). This unrealistic behavior is avoided when group forces are included (see Fig 9). A measure of the group cohesion is given by the the average *distance from the centroid* of the group, defined as

$$\begin{array}{c} \xi \left(t\right)=\frac{1}{n}\sum_{i}^{n}d_{i}\left(t\right), \end{array}$$ (23)

where *d*_*i*_(*t*) is the distance at time *t* of pedestrian *i* from the centroid of the group. This indicator gives a measure of the dispersion of the pedestrians during their motion. The time evolution of *ξ*(*t*) is depicted in Fig 10, for both cases. Without group forces, the group radius oscillates between small values (corresponding to the visitors standing still in front of an artwork) and large values (when people switch from one artwork to the next one). Conversely, the introduction of the group forces effectively keeps the group together, with a radius smaller than 2 m.

### Tuning of the Model

In this section, we study the role of the parameters of the HSFM on the resulting system behavior. Specifically, we consider separately the parameters which affect the computation of: i) the force input, ii) the torque input and iii) the group cohesion term.

#### Force input

**Fig 11:**
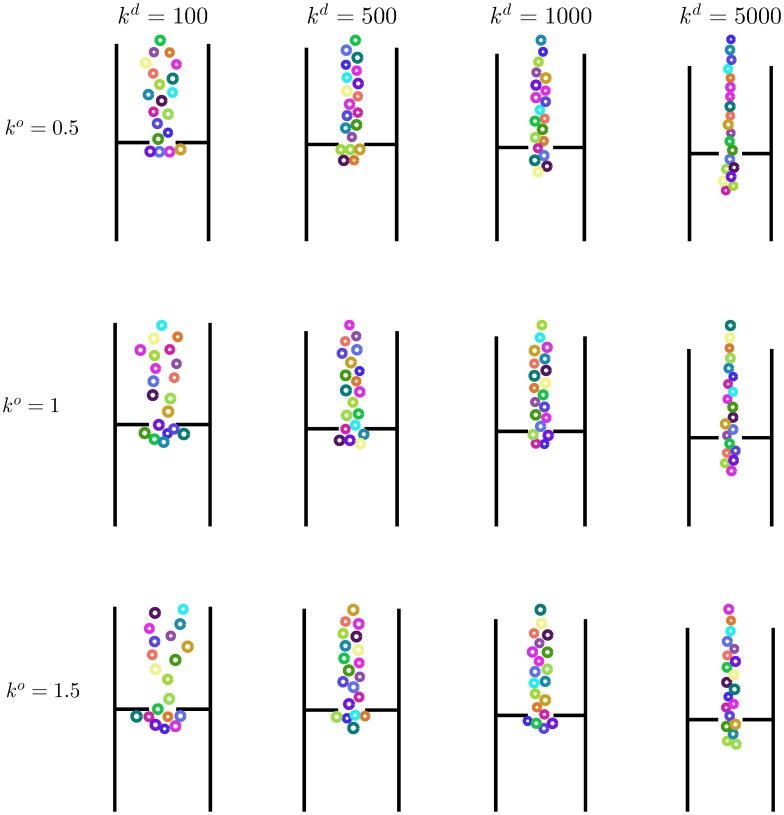
Effect of *k*^*o*^ and *k*^*d*^ on the pedestrian trajectories. A snapshot of the simulation of 20 pedestrians walking in a corridor, for different values of *k*^*o*^ and *k*^*d*^ [kg ⋅ s^−1^].

**Fig 12:**
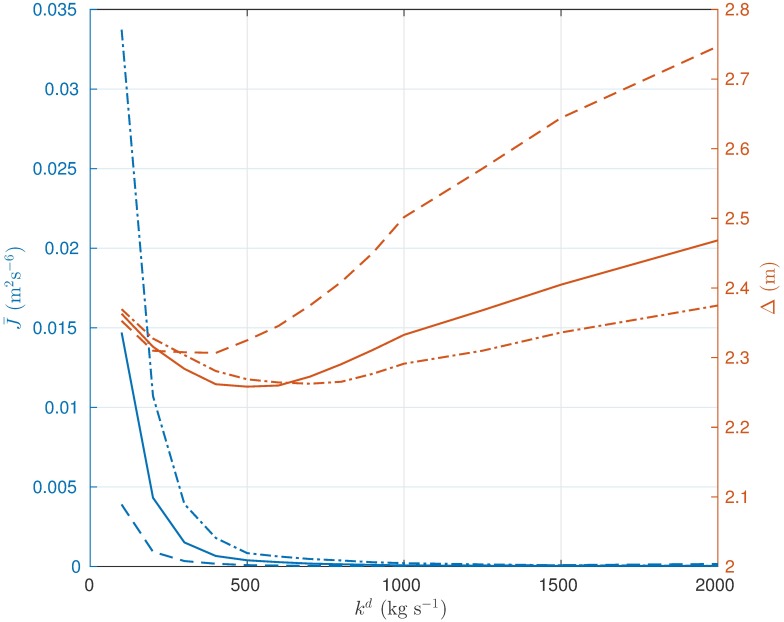
Effect of *k*^*o*^ and *k*^*d*^ on trajectory regularity and distribution of the pedestrians. Average square of the magnitude of the jerk $\bar{J}$ and average distance Δ of a pedestrian from the group centroid for *k*^*o*^ = 0.5 (dashed), *k*^*o*^ = 1 (solid) and *k*^*o*^ = 1.5 (dash-dotted).

The force driving the translational dynamics of the pedestrian depends on two parameters, namely *k*^*o*^ and *k*^*d*^. The first one is a gain that modulates the force acting on the direction orthogonal to the pedestrian’s heading. The second one is a damping coefficient on the speed along the same direction. As a case study representative of the HSFM behavior under most circumstances, the same example, described in Scenario II, involving 20 pedestrian crossing a door in a corridor, is considered (see Fig 4). In this analysis, no group cohesion forces are included. Several simulations have been carried out for different combinations of the parameter values. Fig 11 depicts a snapshot of the simulations taken when the individuals have almost completely crossed the door. By looking at the different configurations of the pedestrians, the following phenomena can be observed. For a given *k*^*d*^, the platoon gets wider as *k*^*o*^ increases, since more authoritative lateral repulsive forces among pedestrians are exerted. Parameter *k*^*d*^ has an even greater impact on the width of the platoon. For a fixed *k*^*o*^, the larger the value of *k*^*d*^, the faster the lateral speed is driven towards zero. As a result, with very high values of *k*^*d*^ the pedestrians tend to arrange in a line. Besides the geometric distribution of the individuals, both parameters have an effect on the smoothness of the generated trajectories. To analyze this feature, 100 simulation runs have been performed, starting from random initial conditions. In Fig 12, two indicators are shown as a function of *k*^*d*^, for different values of *k*^*o*^. The first one is the average square of the magnitude of the jerk $\bar{J}$ as defined in Eq (22), which measures the regularity of the trajectories. The second one is defined as

$$\begin{array}{c} \Delta =\frac{1}{T}\int_{0}^{T}\xi \left(t\right)dt, \end{array}$$

where *ξ*(*t*) is given by Eq (23). It represents the mean distance of a pedestrian from the centroid, averaged over the whole simulation run. The evolution of $\bar{J}$ suggests that the trajectories become more and more regular as *k*^*o*^ decreases and *k*^*d*^ increases. The tuning of parameter *k*^*d*^ has to take into account also the impact that it has on the geometry of the platoon, which in Fig 12 is summarized by the indicator Δ. Too large values of *k*^*d*^ imply a growth of the radius Δ, which, in turns, reflects the tendency of the pedestrians to form a line. Hence, parameter *k*^*d*^ has to be tuned by trading-off these conflicting objectives. Values in a neighborhood of *k*^*o*^ = 1 and *k*^*d*^ = 500 kg ⋅ s^−1^ have been observed to ensure regular trajectories and a realistic geometry of the platoon [10, 37]. Moreover, this choice guarantees very low sensitivity of the indicators $\bar{J}$ and Δ to variations in the model parameters, which suggests robustness of the system behavior within different scenarios.

#### Torque input

**Fig 13:**
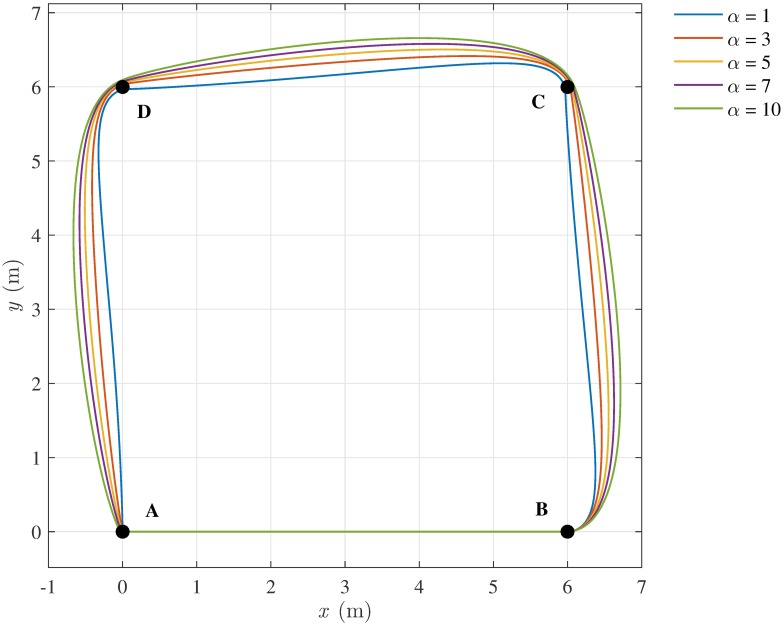
Effect of *α* on the pedestrian trajectories. The path followed by a pedestrian passing through the sequence of way-points A-B-C-D, for different values of the parameter *α*.

The torque controlling the heading dynamics is designed via pole placement, so that the closed-loop system has a desired pair of real poles. In this approach, a major role is played by the pole ratio *α*. The effect of *α* on the resulting trajectory is clearly visible in Fig 13, for the simple case in which a pedestrian goes through four way-points forming a square. Basically, the larger the *α*, the slower is the dynamics of the pedestrian’s heading, which results in larger turning radius. Values of *α* in the range 3-5 seem appropriate for reproducing a realistic path, the resulting curvature dynamics being neither too aggressive (i.e., *α* = 1) nor too loose (i.e., *α* = 10).

#### Group cohesion

The parameters defining the force term which aims at keeping together people belonging to the same group, have a clear physical meaning. This makes their tuning much easier than the previous ones. Parameters *d*^*f*^ and *d*^*o*^ are half of the side length of the desired rectangular region along the forward and orthogonal direction, respectively. Parameters $k_{1}^{g}$ and $k_{2}^{g}$ correspond to the intensity of the cohesion forces acting along the forward and orthogonal direction, respectively. In the simulations presented so far, the following values have been selected: *d*^*f*^ = 2 m, *d*^*o*^ = 1 m and $k_{1}^{g}=k_{2}^{g}=200$ N.

## Conclusions

In this paper, the Headed Social Force Model has been presented. It enhances the traditional Social Force Model with the inclusion of the pedestrians’ heading. A more complex model of the human dynamics is adopted, whose inputs are computed as suitable functions of the force terms resulting from the traditional Social Force Model. An optional force term has been introduced in order to model pedestrians moving together as a group. Numerical simulations show that considering the heading of the individuals improves the realism of the resulting trajectories, in both low pedestrian density scenarios and crowded environments.

The potential of the proposed model opens the door to several future developments. Validation of the human motion patterns predicted by the model on real-world experiments is the subject of ongoing research. Besides assessing the ability of the HSFM to reproduce standard pedestrian behaviors, real data will also be useful to estimate the most significant parameters of the model. In this respect, an interesting topic deserving further investigation is to evaluate how pedestrians’ individual properties (such as gender and age, environmental constraints or social conventions) reflect on the values of the model parameters, in the spirit of the study presented in [38] on aircraft boarding models. Another relevant line of research concerns the generation of the velocities that the pedestrians have to track. The adoption of suitable control schemes may be the first step towards the design of planning strategies to be employed, e.g., for building evacuation, crowd management or group steering.
